# Crystal Structure of the Sema-PSI Extracellular Domain of Human RON Receptor Tyrosine Kinase

**DOI:** 10.1371/journal.pone.0041912

**Published:** 2012-07-25

**Authors:** Kinlin L. Chao, I-Wei Tsai, Chen Chen, Osnat Herzberg

**Affiliations:** 1 Institute for Bioscience and Biotechnology Research, University of Maryland, Rockville, Maryland, United States of America; 2 Department of Chemistry and Biochemistry, University of Maryland, College Park, Maryland, United States of America; Weizmann Institute of Science, Israel

## Abstract

Human RON (Recepteur d’Origine Nantais) receptor tyrosine kinase is a cell surface receptor for Macrophage Stimulating Protein (MSP). RON mediates signal transduction pathways that regulate cell adhesion, invasion, motility and apoptosis processes. Elevated levels of RON and its alternatively spliced variants are implicated in the progression and metastasis of tumor cells. The binding of MSP α/β heterodimer to the extracellular region of RON receptor induces receptor dimerization and activation by autophosphorylation of the intracellular kinase domains. The ectodomain of RON, containing the ligand recognition and dimerization domains, is composed of a semaphorin (Sema), Plexins-Semaphorins-Integrins domain (PSI), and four Immunoglobulins-Plexins-Transcription factor (IPT) domains. High affinity association between MSP and RON is mediated by the interaction between MSP β-chain and RON Sema, although RON activation requires intact RON and MSP proteins. Here, we report the structure of RON Sema-PSI domains at 1.85 Å resolution. RON Sema domain adopts a seven-bladed β-propeller fold, followed by disulfide bond rich, cysteine-knot PSI motif. Comparison with the homologous Met receptor tyrosine kinase reveals that RON Sema-PSI contains distinguishing secondary structural features. These define the receptors’ exclusive selectivity towards their respective ligands, RON for MSP and Met for HGF. The RON Sema-PSI crystal packing generates a homodimer with interface formed by the Sema domain. Mapping of the dimer interface using the RON homology to Met, MSP homology to Hepatocyte Growth Factor (HGF), and the structure of the Met/HGF complex shows the dimer interface overlapping with the putative MSPβ binding site. The crystallographically determined RON Sema-PSI homodimer may represent the dimer assembly that occurs during ligand-independent receptor activation and/or the inhibition of the constitutive activity of RONΔ160 splice variant by the soluble RON splice variant, RONΔ85.

## Introduction

Human RON (Recepteur d’Origine Nantais) receptor tyrosine kinase is the specific cell-surface receptor for Macrophage Stimulating Protein (MSP), a serum growth factor also known as the Hepatocyte Growth Factor-like protein (HGFL). RON, encoded by the *MST1R* gene, is a member of the Class VI receptor tyrosine kinase family (EC:2.7.10.1) along with the proto-oncogene Met receptor tyrosine kinase (Met). The extracellular regions and the cytoplasmic kinase domains of RON and Met share 33% and 64% amino acid sequence identities, respectively [1]. RON is widely expressed in macrophages, epithelial tissues, adenocarcinoma cells, bronchial epithelial cells, granulocytes, and monocytes [2], [3], [4]. The interaction of RON with MSP transduces multiple signaling pathways that regulate cellular morphogenesis, adhesion, invasion and motility [5]. RON is also associated with the MSP-mediated inflammatory activities upon cellular stresses and with innate immune responses to bacterial infections [6], [7], [8]. High levels of RON are detected in patients with ulcerative colitis and deep endometriosis and also in several types of epithelial cancers, implicating RON in tumor progressions and cancer pathogenesis [5], [9], [10], [11]. In addition, alternatively spliced variants of RON promote the metastasises of lung, breast, colon, ovarian, prostate, pancreatic, thyroid and gastric cancers [12], [13], [14], [15], [16], [17], [18], [19], [20]. Thus, RON has become an important target for cancer therapy using anti-RON monoclonal antibodies, small molecule kinase inhibitors, and small interfering RNAs [21], [22], [23].

RON comprises an extracellular ligand binding domain (ectodomain), a single pass trans-membrane segment and a cytoplasmic tyrosine kinase domain. The ectodomain can be subdivided into the N-terminal semaphorin (Sema) domain, a small cysteine-rich Plexins-Semaphorins-Integrins (PSI) motif, and four Immunoglobulins-Plexins-Transcription factor (IPT) domains. Cellular RON is produced as a glycosylated, single chain precursor (Pro-RON), which undergoes a furin protease cleavage at Arg309–Gly310 in the Sema domain prior to its transport from the Golgi to the apical surface of the cell [4], [23]. This disulfide-linked heterodimer is the mature form of RON. RON α-chain contains the N-terminal half of the Sema domain (∼40 kDa) and the β-chain (145 kDa) consists of the second half of the Sema domain, the PSI motif, the four IPT units, the transmembrane region and the cytoplasmic kinase domain. The current model for the MSP-mediated activation of RON begins with the binding of MSP to the receptor, leading to the formation of signaling-competent 2∶2 MSP:RON complex on the cell surface. RON dimerization then promotes the autophosphorylation of the functional tyrosine residues *in trans*, and the up regulation of the intrinsic kinase activity [7], [24], [25]. The phosphorylated kinase domains provide docking sites for cytoplasmic adaptor and signal tranducer proteins to initiate downstream signaling cascades [7], [24]. Some of these signal transduction pathways involve the participations of *ras*/mitogen activated protein kinase (MAPK), phosphatidyl inositol-3 kinase (PI-3K)/Akt, focal adhesion kinase (FAK), and β-catenin proteins [5], [26].

The RON specific ligand, MSP, is a serum protein that stimulates the chemotaxis of mouse peritoneal resident macrophages when exposed to the endotoxin-activated serum [7], [27]. Liver hepatocytes produce a single chain precursor MSP (Pro-MSP), which circulates in blood as biologically inactive scatter factor [28]. Under cellular stress, pro-MSP is cleaved by a type II transmembrane serine proteases, matriptase and hepsin, at Arg483-Val484 to produce the biologically active, disulfide-linked MSP α/β heterodimer [29], [30]. MSP belongs to the plasminogen-like growth factor family along with Hepatocyte Growth Factor (HGF), the specific ligand of Met. The two ligands share 43% amino acid sequence identity [31]. The 50 kDa MSP α-chain (MSPα) contains a N-terminal domain and four Kringle domains. The 30 kDa β-chain (MSPβ) adopts a chymotrypsin-like serine-protease fold [7], [31], [32] but lacks the catalytic triad of serine proteases, and accordingly, is devoid of proteolytic activity (the catalytic triad counterparts in MSP are Gln522, Gln568, and Tyr661) [32]. Pro-MSP exhibits no binding affinity to RON, whereas the mature MSP α/β binds to RON and stimulates the autophosphorylation of tyrosine residues located on the intracellular kinase domain [28]. Both α and β chains of MSP heterodimer are required for RON activation since MSPα or MSPβ alone are incapable of receptor induction [28], [33], [34]. Cell-based binding studies showed similar binding affinities of RON to MSP α/β heterodimer and to MSPβ alone (EC_50_ ∼0.20 nM), indicating that MSPβ contains the high affinity binding site for RON [33]. Surface Plasmon Resonance studies determined a dissociation constant of ∼13 nM between RON full-length extracellular domain and MSPβ [35]. The ability of RON Sema domain to compete with the membrane-bound, full length RON in binding to MSPβ suggested that the MSPβ binding site is localized on the Sema domain [36]. The cell based binding and competition assays also showed that MSPα binds to RON with ∼100-fold lower affinity than that of MSPβ (EC_50_ = 17 nM) [33], [34], [36]. Without MSPβ, MSPα has weak or no affinity for full-length RON, RON Sema, and RONΔ85 splice variant containing only the Sema and PSI domains [16], [34]. However, it remains unknown whether MSPα interacts with any of the RON IPT domains.

In addition to MSP, co-immunoprecipitation and co-localization experiments suggest that RON has other partner proteins. It forms heterodimers with Met, plexin B1–B3, β1 integrin, and epidermal growth factor receptor (EGFR). These interactions control cellular adhesion, migration and invasion processes [37], [38], [39], [40], [41], [42], [43]. For example, RON/EFGR complex can migrate from the cell surface to the nucleus to act as a transcriptional regulator in human bladder cancer cells [44]. RON also associates with several hyaluronan binding proteins. RON/hyaluronidase 2 complex on the cell surface prevents RON’s participation in retroviral transformation of human bronchial epithelial cells [45], [46]. The v6 splice variant of the hyaluronan receptor CD44 associates with RON/MSP during the migration of human colon adenocarcinoma cells, and another hyaluronan receptor, RHAMM (Receptor for Hyaluronic Acid-Mediated Motility) was co-localized with RON at the apical surface of ciliated cells in response to oxidative stress [47], [48].

Despite a wide range of cellular responses regulated by RON, the basic mechanisms by which MSP and other protein partners mediate RON’s activity are unknown at atomic detail. Here, we report the crystal structure of RON Sema-PSI domains at 1.85 Å. The structure reveals unique MSP specificity determinants. It also suggests possible mechanisms for the ligand-independent RON dimerization, which occurs at high RON expression levels and with RONΔ160 splice variant, present in a wide range of human tumors and tumor-derived epithelial cell lines [23], [49].

## Materials and Methods

### Cloning, Expression and Protein Purification

The human *MST1R* gene was amplified from pMSCVneo-hRON-2HA, (kindly provided by Dr. Pamela A. Hankey, Penn State University) and was ligated into the BglII/AgeI digested pMT/BiP/V5-HisA vector for the secreted RON Sema-PSI-IPT_1_ production in the *Drosophila* Expression System (Invitrogen). The furin cleavage site in the RON Sema domain (KRRRRGA) was mutated to a thrombin cleavage site (KLVPRGS). The recombinant RON Sema-PSI-IPT_1_ protein spans residues Glu25–Glu683 along with two N-terminal residues (Arg23 and Ser24) and two C-terminal residues (Thr684, Gly685) followed by a His_6_-tag, (His686–His691), derived from the expression vector. Sequencing revealed the presence of the mutation Arg322Gln due to single nucleotide polymorphorism. *Drosophila melanogaster* Schneider 2 (S2; Invitrogen) cells were cotransfected with the RON expression vector and pCoPuro, and the stable transfectants resistant to puromycin were selected. Clonal selection of stable transfectants was conducted to obtain clones with high protein expression levels. RON protein, secreted into the conditioned serum free media (HyClone SFX), was detected by Western analysis using the C-terminal specific Penta-His monoclonal Antibody (Qiagen). For large-scale preparation, stable S2 cells were grown in shaker flasks at 28°C and protein production was induced by the addition of 0.6 mM CuSO_4_. After 4–5 days, S2 cells were removed by centrifugation and the conditioned medium was applied directly onto a Chelating Sepharose Fast Flow column (GE Health Sciences) (Lehr et al., 2000). His_6_-tagged RON protein was eluted with 50 mM Tris-HCl, (pH 8.0) containing 50–500 mM imidazole. RON Sema-PSI-IPT_1_ was further purified by 40% and 80% ammonium sulfate precipitation steps. Sephacryl S200 size exclusion chromatography equilibrated in 50 mM Tris-HCl, pH 8.0, 0.1 M NaCl, 0.5 mM EDTA (GE Health Sciences) was used to remove ammonium sulfate and contaminants. Protein concentration was determined using a calculated extinction coefficient value of 44,485 M^−1^ cm^−1^ at 280 nm. The yield was ∼1.7 mg purified protein per 1 L media. The matrix-assisted laser desorption time-of-flight (MALDI-TOF) mass spectrometry analysis showed the molecular mass of 77,114±91 Da, ∼5206 Da higher than the calculated molecular mass of 71,908 Da, consistent with the five predicted N-glycosylation sites, four in the Sema and one in the IPT_1_ domain. Assuming uniform glycosylation, the average sugar mass per site is ∼1,041 Da, within the range for a simple 5-unit biantennary carbohydrate reported to be commonly synthesized in *Drosophila* S2 cells [50]. The RON Sema-PSI-IPT_1_ was cleaved with thrombin at 1000∶1 substrate:enzyme molar ratio for 16 hours at 22°C to obtain a disulfide-linked RON α/β heterodimer. Thrombin was removed from the proteolysis reaction by affinity chromatography using Benzimidine Sepharose resin (GE Health Sciences). The mature RON α/β migrated on the SDS-PAGE as a single chain under non-reducing condition, while it ran as the 30 kDa α- and 50 kDa β-chains under reducing conditions.

### Crystallization, Data Collection, and Structure Determination

Crystals were obtained at room temperature by the hanging drop or sitting drop vapor diffusion methods. Equal volumes of 7.1 mg/mL RON Sema-PSI-IPT_1_ sample and mother liquor containing 0.1 M sodium acetate (pH 4.6), 19% (v/v) polyethylene glycol (PEG) 4000, 0.2 M ammonium sulfate (derived from the Hampton Crystal Screen I condition 20) were dispensed, and the drops were equilibrated against the reservoir solution. Both the single chain and thrombin-cleaved RON Sema-PSI-IPT_1_ formed crystals under identical condition. For data collection, plate-like RON Sema-PSI-IPT_1_ crystals were transferred to mother liquor supplemented with 30% (v/v) glycerol and flash-cooled at 100° K in liquid propane cooled in liquid nitrogen. Diffraction data for the single chain RON Sema-PSI-IPT_1_ were collected at the General Medicine and Cancer Institutes Collaborative Access Team (GM/CA-CAT) micro-beamline at the Advanced Photon Source (Argonne National Laboratory, Argonne, IL), which was equipped with a MARmosiac CCD detector. The data were processed with the XDS (Table 1) [51]. Diffraction data for the thrombin-cleaved RON crystal were collected in-house and processed with d*TREK [52]. The crystals of the single chain and cleaved RONs were isomorphous belonging to the space group C2 with one molecule per asymmetric unit and solvent content of 55.3%.

**Table 1:** Data collection and refinement statistics.

*Data collection*	
Space group	C2
Resolution (Å)	19.3−1.85 (1.90–1.85)
Cell dimension (Å)	a = 106.4, b = 73.3, c = 87.9, α = γ = 90, β = 97.4
Wavelength (Å)	1.0332
No. molecules in the asymmetric unit	1
No. observed reflections	144,495
No. unique reflections	51,510
Completeness (%)^a^	89.9 (63.7)
Multiplicity	2.8 (2.0)
*R*_merge_ b	0.069 (0.44)
<I/σ>	10.46 (1.47)
*Refinement*	
Resolution range (Å)	19.3−1.85
No. of reflections	48,882
Completeness (%)	90.0
*R*_factor_ c/*R*_free_ d	0.191/0.232
No. protein atoms	3845
No. of waters	358
No. of sugar units	4
No. of ethylene glycol, PEG fragment, acetate, sulfate, glycerol	5, 1, 6, 2, 4
*Root Mean Square Deviation from ideal geometry*	
Bond length (Å)	0.012
Bond angles (°)	1.55
*Ramachandran plot (%)*	
Allowed	99.5
Disallowed	0.5

a The values in parentheses are for the highest resolution shell, 1.85 Å.

b *R_merge_*  = ∑*_hkl_* [(∑*_j_* | *I_j_* – < *I* > | )/∑*_j_* | *I_j_* | ].

c *R_factor_*  = ∑*_hkl_* | |*F_o_*| – |*F_c_*| |/∑*_hkl_* |*F_o_*|, where *F_o_* and *F_c_* are the observed and calculated structure factors, respectively.

d *R_free_* is computed from randomly selected 2,628 reflections and omitted from the refinement.

The structure was determined by Molecular Replacement using PHASER [53] as implemented in CCP4, with the Met Sema domain (PDB entry code 2UZX) as the search model [54]. Model rebuilding and structure refinement were carried out using the programs Coot [55], and REFMAC5 [56]. Water molecules were assigned using peaks in the *F*_o_ – *F*_c_ difference Fourier map with electron density >3σ as the acceptance criteria. As the diffraction resolution of the single chain RON crystal was superior to that of the thrombin-cleaved RON Sema-PSI (1.85 Å and 2.5 Å resolution, respectively), the refinement results are provided for the intact RON structure (Table 1). The coordinates and structure factors have been deposited in the Protein Data Bank (entry code 4FWW).

Molecular interfaces were calculated using the PISA server [57] and figures were prepared with PyMOL (DeLano Scientific, CA), MOLSCRIPT [58] and RASTER3D [59].

## Results and Discussion

### Characterization of the Purified RON and RON in the Crystals

The purified RON Sema-PSI-IPT_1_ has a molecular mass of 77,114±91 Da, consistent with the five predicted glycosylation sites. The electron density map, however, accounted only for the Sema and PSI domains. SDS-PAGE analysis of the crystals suggested degradation of the protein during crystallization. The mass spectrometry analysis of RON crystals revealed a major peak of 65,616±235 Da. Crystal packing positions Val42 as the first residue seen in the electron density map. Val42 is located in a highly crowded environment with no space to accommodate the preceding amino acids. The lower than expected molecular weight and the arrangement in the crystal suggest a proteolytic cleavage of the 17 N-terminal residues. Moreover, western analysis of RON crystals showed a truncated protein that was not recognized by the C-terminal specific Penta-His antibody, indicating additional loss of C-terminal residues. Thus, a second proteolytic cleavage site occurred in the IPT_1_ domain. Accounting for the loss of the 17 N-terminal residues, the difference between the calculated and experimentally determined molecular mass implies the degradation of approximately 75 of the 115 IPT_1_ residues. No electron density was associated with these remaining IPT_1_ residues and accordingly, they were not modeled. The *Drosophila* S2 cells produce at least four acid active cathepsins [60], and trace amounts of contaminating proteases in the RON Sema-PSI-IPT_1_ preparation could cleave the protein at the low pH (4.6) of the crystal growth solution. In contrast, the RON Sema-PSI-IPT_1_ protein remained intact when stored at pH 8.0. However, the loss of the C-terminal residues of IPT_1_ during crystallization may be related to the trypsin-sensitive Lys632-Lys633 peptide bond of IPT_1_ [49]. Ma and colleagues concluded that the susceptibility of RON IPT_1_ to the cell-associated trypsin-like proteases regulates RON mediated tumorigenic activities in epithelial cells.

### Structure Determination

**Figure 1:**
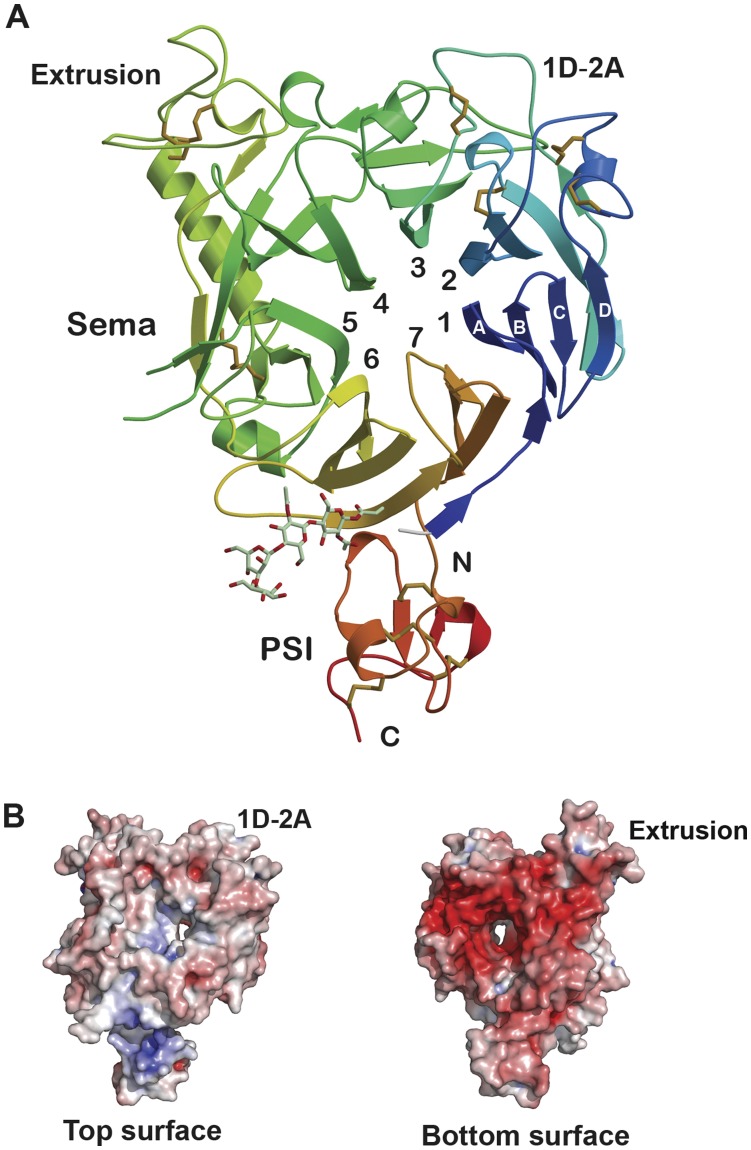
Structure of the Sema-PSI domains of human RON receptor tyrosine kinase. (A) Ribbon representation of RON Sema-PSI, viewed down the β-propeller, with the color ramped from blue at the N-terminus to red at the C-terminus. The blades are numbered and the antiparallel β-strands of each blade are labeled A–D from the inner to the outermost strands. Disulfide bonds are shown in red and the N-linked oligosaccharide is shown as stick models with the atomic color scheme: Green – carbon, red – oxygen, blue – nitrogen. (B) Surface rendering of the top and bottom surfaces of RON Sema-PSI, represented by electrostatistic potential from red (−10 k_b_T/e_c_) to blue (10 k_b_T/e_c_), as generated by PyMol. The left panel represents the top surface using the same orientation as in (A). The right panel corresponds to the opposite or bottom β-propeller surface.

The Molecular Replacement yielded a single solution for the RON Sema domain with the rotation and translation function Z-scores of 10.8 and 9.6, respectively. However, various Molecular Replacement searches with the Met PSI domain did not yield a correct solution. The initial model for the RON PSI domain was obtained by manual fitting of the Met PSI domain into the electron density map, and then modifying the main chain and side chains as required. There is one RON sema-PSI molecule per asymmetric unit. The loop that encompasses the thrombin cleavage site was disordered in both the single-chain and thrombin-cleaved RON Sema-PSI proteins. Overall, the RON Sema-PSI model contains amino acids Val42–Pro568 (Figure 1A) except for nine Sema residues encompassing the disordered thrombin cleavage site (Leu306–Gly314) and four loop residues (Asp355–Pro358). Of the four predicted RON Sema N-glycosylation sites (Asn66, Asn419_,_ Asn458, and Asn488), only the Asn488 N-glycan was visible in the electron density map, consistent with a branched biantennary oligosaccharide Manα(1,3)Manβ(1,4)GlcNAcβ(1,4)GlcNAc.

### Overall Structure

**Figure 2:**
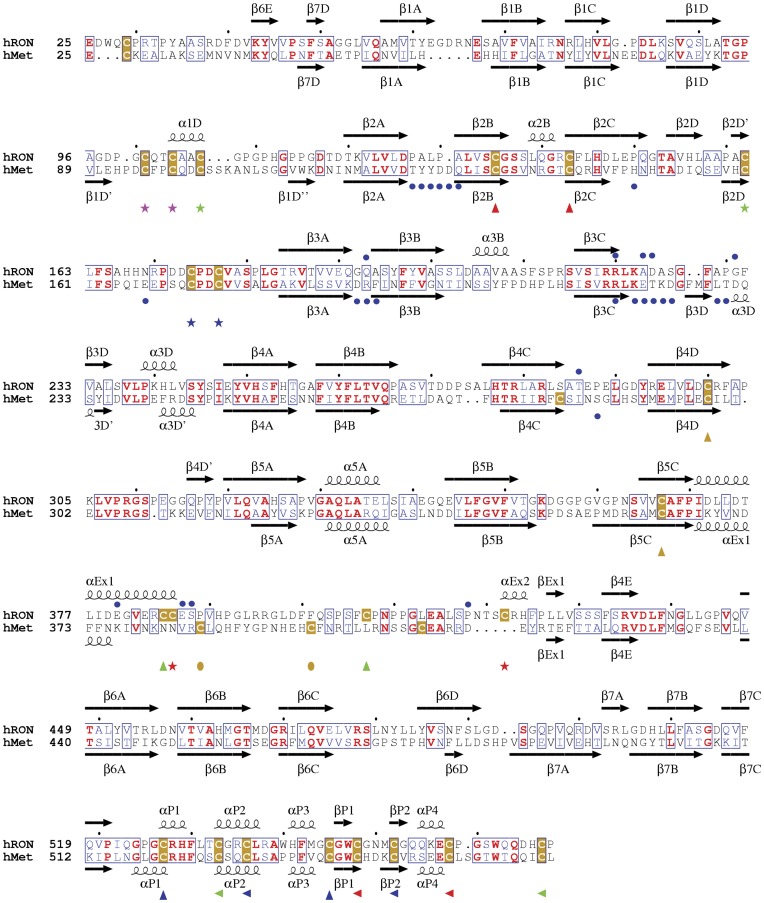
Structure-based sequence alignments of human RON and Met Sema-PSI. Residues are colored as follows: Identical residues (red), and conservatively replaced residues (blue) are boxed. Cysteines are colored gold. Matching colored symbols indicate pairs of cysteines that form disulfide bonds. Secondary structure units of RON and Met are labeled. The blue dots above the RON Sema residues indicate amino acids at the symmetry-related RON Sema-Sema interface as discussed in the text. The blue dots below the Met Sema sequence show residues that contact the HGFβ ligand (PDB code 1SHY) (Stamos et al., 2004). This figure was prepared with ESPript (espript.ibcp.fr/Espript/).

The RON Sema domain adopts the seven-bladed β-propeller fold, found in Met, semaphorins and plexin receptors (Figure 1A) [61], [62], [63]. The DALI program [64], [65] identified the Met Sema domain (PDB code 1SHY) as the closest to the RON Sema domain, with Z = 41.1 and root mean square deviation (RMSD) of 3.1 Å for 462 Cα atoms. More distant structural homologues of RON Sema include the plexin receptors and semaphorins [66]. RON Sema contains hallmark features of the Sema-type β-propeller. These features include a large insertion, termed extrusion, in the 5^th^ blade and deviations from four antiparallel β strands, characteristic of the individual β-sheets in the β-propeller fold [67], [68], [69]. In RON and Met Sema domains, blade 5 of the β-propeller contains three β-strands (strands A–C) and blades 4 and 6 contain five β-strands (strands A–E) with strand A being the innermost β-strand at the center of β-propeller (Figure 1A). The N-terminal residues (Val42–Tyr44) provide the 5th β-strand (β6E) of blade 6. The extrusion regions in blade 5 vary both in sequence and length in different Sema-type β-propellers, ranging from 57 residues in Met to 77 residues in Sema4D [68]. In RON, the 62-residue insertion (Pro370–Ser432) is located between β-strands 5C and 4E (Figure 2), and it forms a 16-residue helix (αEX1), followed by a long loop that packs tightly against the outermost 4E (Figure 1A). Structural integrity of this long loop is maintained by two adjacent disulfide bonds (Cys385–Cys407 and Cys386–Cys422) and by stacking of aromatic groups (Phe400 of the extrusion and Tyr245 of α-helix 3D of the core Sema domain). Electrostatic potential analysis showed that the top surface of the β-propeller barrel, corresponding to loop segments connecting the β-strands BC and DA, and the sides of the barrel are neutral. In contrast, the bottom surface of the β-propeller barrel, corresponding to loop segments connecting the AB and CD β-strands is negatively charged (Figure 1B). The pronounced negatively charged surface suggests interaction with a positively charged region of a counterpart protein.

A small, cysteine-rich PSI motif follows the Sema domains of both RON and Met receptor tyrosine kinases. PSI modules, found in the extracellular domains of over 1,600 structurally and functionally related receptor proteins, serve as hinges to orient the preceding and ensuing domains for proper receptor-ligand interactions [70]. The PSI motifs of RON and Met adopt a cysteine-knot fold consisting of two small antiparallel β-sheets and four short α-helices (Figure 1A). RON PSI contains 8 conserved cysteines, which form four disulfide linkages (Cys527–Cys545, Cys536–Cys552, Cys548–Cys558, and Cys533–Cys567 in RON sequence). A DALI alignment of the RON and Met PSI domains yielded Z = 8.0 and RMSD = 1.5 Å for 44 paired Cα atoms. RON PSI motif shows a negatively charged surface on one side as it extends from RON Sema’s bottom surface (Figure 1B, right panel), while positive charged residues populate the opposite surface of the PSI motif (Figure 1B, left panel). The interdomain contact area between the RON Sema and PSI domains embeds ∼385 Å^2^ surface area. The small interaction surface is consistent with a flexible module that mediates the conformational transition of multi-domain cell surface receptors.

### Comparison with Met Sema-PSI Structure

**Figure 3:**
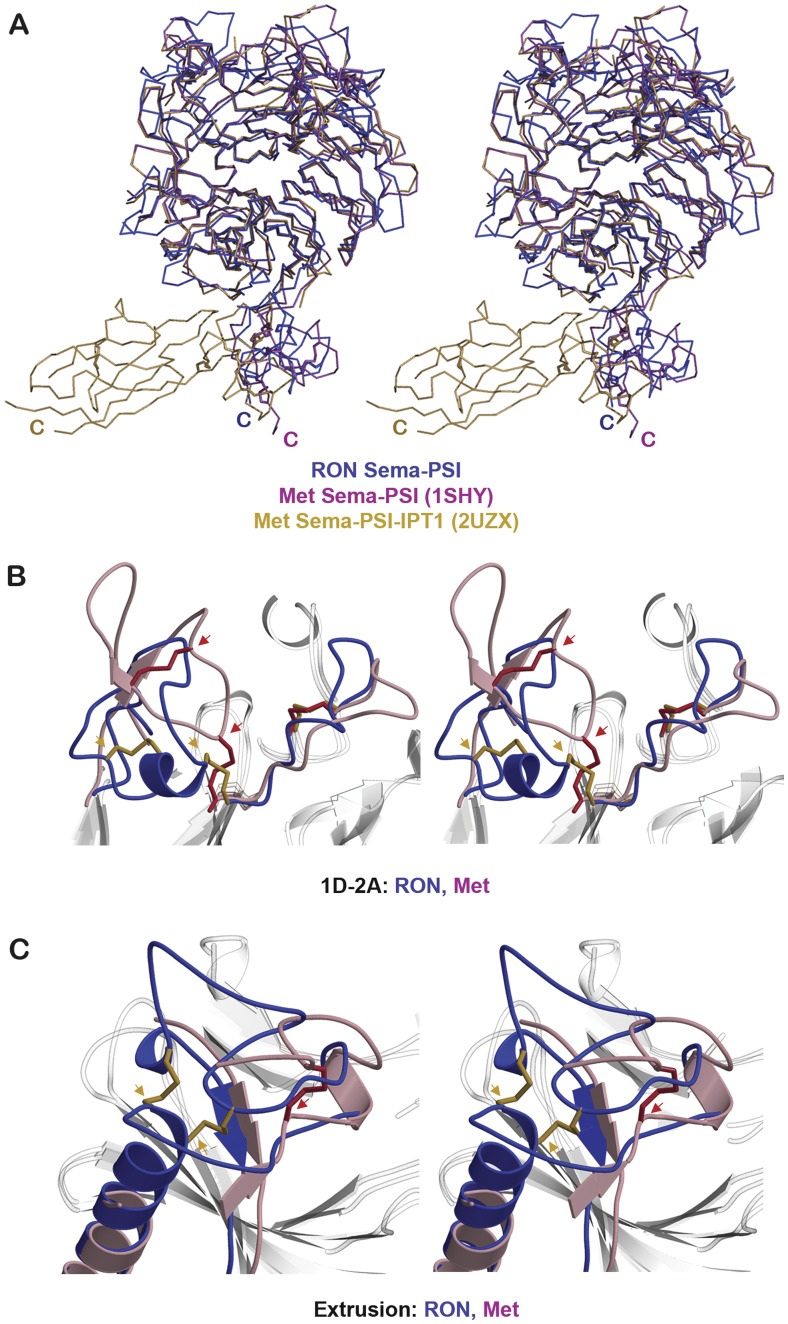
Comparison of RON and Met structures. (A) Stereoscopic representation of superposed RON Sema-PSI (blue) and Met Sema-PSI (PDB entry codes 2UZX (gold) and 1SHY (pink) structures, viewed down the β-propeller as in Figure 1A. The Sema domains were superposed. (B) The superposed RON and Met, highlighting the loop connecting β-strands 1D and 2A and (C) highlighting the extrusion regions. Disulfide bonds of RON Sema are colored gold and those of Met Sema (PDB code 1SHY), red. The gold and red arrows highlight the locations of alternative disulfide linkages in RON and Met, respectively.

The RON and Met extracellular domains share ∼35% sequence identity. A structure-based sequence alignment of RON and Met Sema-PSI shows that, by and large, the secondary structural elements are conserved (Figure 2). The loops connecting the secondary structure elements are less conserved and contain multiple insertions and deletions. RON Sema loops contains α-helices that are absent in Met (α1D, α2B, α3B and αEx2; Figure 2), while the Met Sema loops have two β-strands that are absent in RON (β1D’ and β3D; Figure 2). The superposed structures show that the core β-sheets of the RON and Met β-propellers are well aligned but many of the surface loops adopt different conformations (Figure 3).

RON and Met Sema domains contain 15 cysteine residues that form disulfide linkages (Figure 1A, 2). Three disulfide bonds are conserved (Cys135–Cys143, Cys300–Cys367, and Cys174–Cys177 in RON and Cys133–Cys141, Cys298–Cys363 and Cys172–Cys175 in Met). They link the intra β-strands 2B and 2C, the inter blade β-strands 4D and 5C, and a 20 residue loop connecting blades 2 and 3. However, four other conserved cysteine residues (Cys101, Cys104, Cys107 and Cys162 in RON Sema and Cys95, Cys98, Cys101 and Cys160 in Met Sema) form two different pairs of disulfide bonds (Figure 2, 3B). In RON, the linkages are between Cys101 and Cys104 located on the α-helical turn containing loop that connects β-strands 1D and 2A, and between Cys107 on the same loop and Cys162 located on β-strand 2D (Figure 3B). Alternative disulfide pairings have been reported for the analogous Met Sema loop in the Met/HGFβ structure (Cys95–Cys101 and Cys98–Cys160), but not in the Met/InlB structure where this loop was disordered and Cys160 was unpaired [63], [71]. The alternative disulfide bond and shorter 1D–2A loop of the RON Sema domain lead to a compact loop, whereas a longer loop in Met Sema lacks α-helical turn and folds into a flexible loop that extended above the core of β-propeller (Figure 3B) (Stamos et al., 2004). One more conserved cysteine in RON and Met Sema is located near the respective N-termini (Cys29 in RON and Cys26 in Met) (Figure 2). RON Cys29 and Met Cys26 are predicted to form an interdomain disulfide bond with the conserved cysteine in IPT_1_ domain (RON Cys590 and Met Cys584) [63]. In the RON Sema-PSI structure, Cys29 has been removed by proteolysis and Cys590 is located within the disordered RON IPT_1_ fragment. The putative Met Cys26–Cys584 inter-domain disulfide bond was also not observed in either Met/HGFβ or Met/lnlB structures [63], [71].

**Figure 4:**
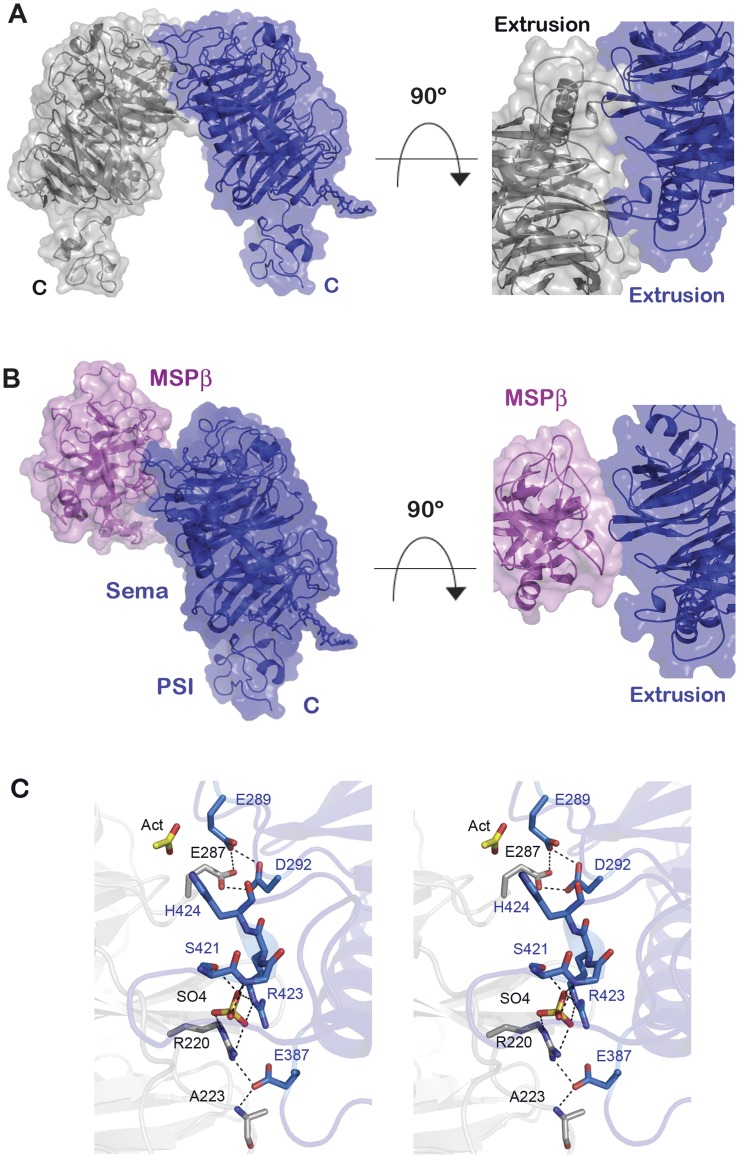
Crystal packing generates a RON homodimer interface that overlaps with the putative MSPβ binding site predicted based on the Met/HGF structure. (A) Left panel: Surface and ribbon representations of symmetry-related RON Sema-PSI molecules. Right panel: Close-up view of the interface and the molecules rotated by ∼90°. (B) Surface and ribbon representation of the modeled RON Sema-PSI/MSPβ complex derived based on the free MSPβ (PDB code 2ASU) and RON Sema-PSI structures superposed onto the structure of Met Sema-PSI/HGFβ (PDB entry 1SHY). The molecular surfaces of RON Sema-PSI (blue) and MSPβ (pink) are shown in transparent colors and secondary structural elements are shown in ribbon representation. (C) Stereoscopic representations of the RON Sema homodimer interface residues generated by crystal packing. The two subunits are colored gray and sky blue. Selected amino acids are colored in the atomic color scheme: red, oxygen; blue, nitrogen; dark yellow, sulfur; bright yellow, acetate carbon.

Two more RON Sema disulfide bonds are located on the large extrusion region (Cys385–Cys407 and Cys386–Cys422). These cysteine residues are not conserved in Met Sema. Instead, the extrusion of Met Sema contains a single disulfide bond (Cys385–Cys397) and an unpaired Cys409 in the disordered loop (Figure 2 and 3C) [63], [71]. Another non-conserved Cys282 in Met Sema is positioned at the end of β-strand 4C near the extrusion region. In all, the alternate disulfide bonding patterns in the 1D–2A loop and the extrusion regions of RON and Met Sema domains define specificity determinants, which allow RON and Met receptors to interact exclusively with either MSP or HGF, respectively.

As reported earlier, when the two available structures of Met are compared, the superposed structures of the Met/HGFβ and Met/InlB complexes reveal different orientations assumed by the Met PSI with respect to the aligned Sema domains [71]. The C-termini of the Met PSI in these structures are displaced by ∼15 Å and are rotated by ∼60° with respect to a common axis defined by the region linking the Sema and PSI domains. The RON PSI module adopts yet another orientation (Figure 3A). The RON PSI is flanked on one side by the Met PSI from the Met/HGFα complex with ∼8 Å displacement, and on the other side by the Met PSI from the Met/InlB complex with ∼10 Å displacement (Figure 3A). Similarly to Met, the conserved Gly524 and Gly526 located at the Sema-PSI linker region modulate the relative orientation of RON PSI (Figure 2). Moreover, both structures of Met complexes and RON Sema-PSI structure lack the disulfide bond predicted to link the disordered/degraded N-terminal region with the IPT_1_ domain. The relative orientations of the Sema, PSI, and IPT_1_ domains might still be different in the presence of this interdomain disulfide linkage.

The ability of RON and Met ectodomains to adopt multiple interdomain orientations may play critical roles in selective ligand binding and receptor dimerization. For instance, the RONΔ160 splice variant, lacking the 103 residue long IPT_1_ domain, readily forms dimers in the absence of MSP and displays constitutive phosphorylation activity [49]. In the absence of IPT_1_ domain, the adoptable PSI hinge may provide a mechanism for reorientation of the remaining RON ectodomains that allow MSP-independent receptor dimerization and concomitant juxtaposing of the intracellular kinase domains for autophosphorylation.

### Proteolytic Maturation

Among the semaphorin superfamily, RON and Met contain a furin protease cleavage site in a loop connecting β-strands 4D and 5A of Sema domain. In contrast, the semaphorins and plexin receptors lack a furin recognition site [68]. The proteolytic maturations of RON and Met are required for their signal transduction activities [4]. In addition, the lysine and arginine-rich furin cleavage site in RON has been identified as one of two consensus nuclear localization signal sequences that may play a role in the transcriptional regulatory function of RON/EGFR complex in human cancer cells [44]. We have determined the structures of single chain and thrombin-cleaved RON Sema-PSI, hoping to gain structural insight into the functional role played by this specific cleavage event. Two structures are identical within the accuracy of the data, and the loop is disordered in both cases. This suggests that the proteolytic maturation of Pro-RON into α and β chains does not induce conformational changes in the RON Sema-PSI; rather it may be involved in MSP-induced homodimerization, and/or facilitate the weak interaction with the MSP α chain. Similarly, the counterpart 9-residue surface-exposed loop of Met is disordered in Met/HGFβ and Met/InlB structures [63], [71]. The equivalent 4D–5A loop without the consensus furin cleavage site in the semaphorins is involved in homodimerization, but not in plexin receptors [62], [67]. Thus, the structural basis for the mechanism of proteolytic maturation, required for RON and Met receptor activation, remains unclear.

### Ligand-independent RON Dimerization

In addition to the MSP-mediated RON receptor activation, ligand-independent RON dimerization and constitutive phosphorylation activity have been observed in numerous cancer and tumor cells which over expressed full length RON receptor and expressed the RONΔ160 splice variant [20], [72], [73]. RON intermolecular interactions generated by the crystal packing reveal a potential mode of ligand-independent dimerization, mediated by the Sema domain (Figure 4A). This is the most extensive crystallographic related RON Sema-Sema interface with ∼960 Å^2^ embedded surface area, a rather large interface for typical crystal contacts [74]. Therefore, this crystal-generated interface may have a functional role at the cellular level. Multiple electrostatic interactions between the bottom surface loops of blades 3–4 and the edge residues of the extrusion region are involved, and these are repeated twice due to the crystal 2-fold symmetry axis. Two striking networks stand out within this dimer interface. First, Glu387 forms an intermolecular salt bridge with Arg220, and the carboxylate group of Glu387 also interacts with the NH of Ala223 of the neighboring molecule (Figure 4C). The guanidinium groups of Arg220 and Arg423 of the partner Sema interact with an interface sulfate ion (present in the crystallization solution). The positioning of the sulfate ion is further stabilized by the hydrogen bonding with the hydroxyl group of Ser421 and by the main chain NH groups of Cys422 and Arg423. The arrangement of this sulfate-binding site appears optimal for accommodating a phosphoryl group on Ser421, although the physiological phosphorylation state of this residue is unknown.

A second intermolecular electrostatic cluster at the crystallographic RON Sema-PSI dimer interface comprises three carboxylate groups; two from one subunit (Glu289 and Asp292) and the third from the second subunit (Glu287) (Figure 4C). This particular type of proton sharing interaction between carboxyl-carboxylate groups is favorable only at pH below 6 (Sawyer and James, 1982**)**, consistent with the acidic condition (pH 4.6) used to obtain the RON Sema-PSI crystals. Multitude secondary and tertiary shells of interactions support the formation of both electrostatic clusters. Finally, an acetate ion (pH buffering component of the crystals) is located on a special crystallographic 2-fold symmetry position, bridging two His242 imidazole groups, albeit at somewhat remote distances (3.4 Å). The pH-dependent intermolecular interactions, described above, suggest that ligand-independent homodimerization of RON may play a functional role in the acidic extracellular microenvironments often associated with tumors and under other cellular acidosis conditions [75], [76].

This crystallographically observed RON Sema-PSI homodimer, generated by a Sema-Sema interface, might pertain to the mechanism of ligand-independent constitutive activity of RONΔ160 splice variant and its inhibition by RONΔ85 [16]. RONΔ160, lacking the 103-residue IPT_1_ domain, is a cell surface receptor that readily forms homodimers and is constitutively active in the absence of MSP. RONΔ85 splice variant, on the other hand, is a soluble protein comprising only the Sema, PSI, and 64 amino acid residues of IPT_1_ domain. The addition of RONΔ85 reduced the levels of phosphorylated RONΔ160 as well as those of phosphorylated downstream signaling molecules, ERK1/2 and Akt, in a dose-dependent manner [16]. The co-immunoprecipitation experiments revealed a direct association between RONΔ160 and RONΔ85 molecules, and RONΔ160 dimerization was lower in cells treated with RONΔ85 [16], [20]. MSP did not prevent the RONΔ85 inhibition; thus, the dominant negative effect appears to be a direct consequence of RONΔ85 binding to the membrane-bound RONΔ160 [16]. Ma and colleagues suggested the Sema-Sema interaction between RONΔ85 and RONΔ160 as the possible mechanism of inhibition, perhaps employing the Sema-Sema interface observed in the RON Sema-PSI structure (Figure 4A).

The full length RON also exhibits ligand-independent dimerization at high receptor density, which may be responsible for its constitutive activity in tumors [14], [40], [72], [73]. RONΔ90 splice variant, comprising Sema, PSI and 70 amino acids of IPT_1_, was shown to inhibit the MSP-induced RON phosphorylation activity and to attenuate the basal constitutive activity of RON in the absence of external MSP. RONΔ90, found in several glioblastoma cell lines, blocked both the MSP-induced migration and random motility of these cells [14]. Analogous to the interaction between RONΔ85 and RONΔ160 splice variants, we propose that RONΔ90 splice variant may sequester the full length RON as an inactive dimer using the mode of homodimerization seen in the crystals, thus exerting an antagonistic effect on cell migration.

In this crystal homodimer, the PSI motifs extend from their respective Sema domains in the same direction as expected for membrane-anchored receptors (Figure 4A). Approximately 50 Å separates the C-termini of the PSI domains, a reasonable distance that can be bridged by the IPT domains to bring together two membrane-spanning segments so that the intracellular kinase domains can interact and undergo constitutive autophosphorylation in trans.

RON Sema domain was identified as the high affinity binding site for MSPβ [33], [36]. We have mapped the high affinity MSPβ binding site on the RON Sema domain, based on the Met Sema-PSI/HGFβ structure and the structural homologies between the RON and Met receptors and their MSP and HGF ligands (Figure 4B). The model shows a region of the RON homodimer interface overlapping with a same region of RON Sema predicted to bind to MSPβ. The overlap between the binding regions lends support to our proposal that the crystallographically observed mode of RON Sema homodimerization represent the *in vivo* ligand-independent, constitutively activated RON homodimer. Similar modes of protein-protein interactions occur in the semaphorins and plexin receptors. That is, in semaphorins and plexins, the extrusion region of one Sema subunit interacts with a second homodimer subunit, or with the ligands or co-receptors [61], [62], [66], [67]. For example, using the same interface, plexin A2 dimer undergoes a partner switch to accommodate Sema6A dimer ligand, forming a 2∶2 signaling complex [62]. Although the arrangements of the RON, semaphorin, and plexin homodimers differ, all interfaces engage the extrusion region present in all Sema domains but structurally unique in each family member [62], [67], [69].

We note a second crystal packing interaction between symmetry-related RON Sema-PSI molecules involving a much smaller embedded surface area (∼390 Å^2^), mediated by hydrophobic interactions. Top surface loops connecting β-strands 4E–6A, 6B–6C and 6D–7A along with the N-glycans linked to Asn488 of RON Sema participate in formation of this Sema-Sema interface (data not shown). In this homodimer arrangement, the RON PSI motifs also extend from the respective Sema domains in the same direction and their C-termini are separated by ∼15–24 Å. The IPT domains of this dimer can make molecular contacts along the stalk of RON’s ectodomain. However, this interface only formed because the N-terminus of RON Sema had undergone proteolysis. Formation of such dimer would be blocked in the presence of the N-terminal residues.

In summary, the structure of RON Sema-PSI provides new insights into the features that define the MSPβ specificity and the possible mechanism of ligand-independent RON receptor activation. Analysis of RON mode of homodimerization and comparison with the semaphorins and plexin receptors suggests that all Sema-type proteins employ homodimerization interfaces that overlap with the ligand binding interfaces as a mechanism to regulate their signaling activities.
